# Refactoring the Conjugation Machinery of Promiscuous Plasmid RP4 into a Device for Conversion of Gram-Negative Isolates to Hfr Strains

**DOI:** 10.1021/acssynbio.0c00611

**Published:** 2021-03-22

**Authors:** Jillian Silbert, Victor de Lorenzo, Tomás Aparicio

**Affiliations:** † Systems and Synthetic Biology Program, Centro Nacional de Biotecnología (CNB-CSIC), Campus de Cantoblanco, Madrid, 28049, Spain

**Keywords:** *Pseudomonas*, Hfr, RP4, conjugation, genomic transfer

## Abstract

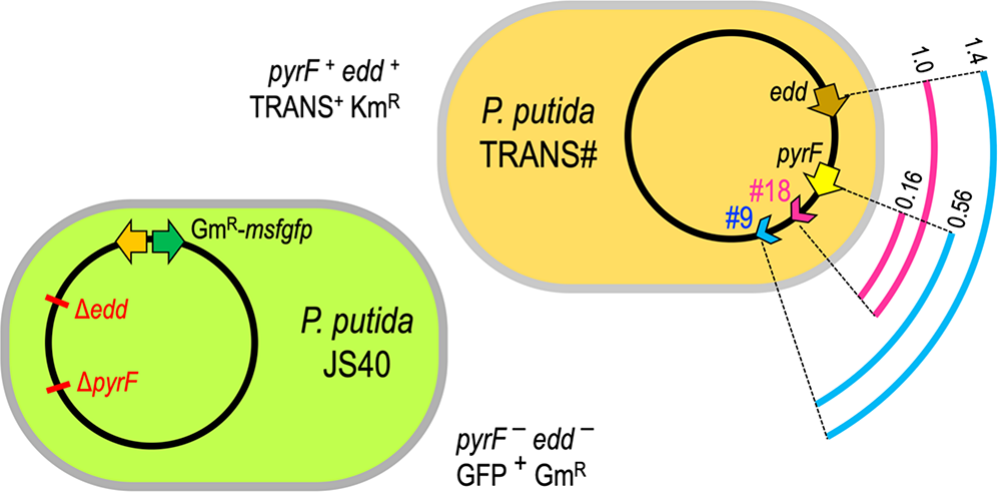

Chromosomal exchange and subsequent recombination of the cognate DNA between bacteria was one of the most useful genetic tools (e.g., Hfr strains) for genetic analyses of *E. coli* before the genomic era. In this paper, yeast assembly has been used to recruit the conjugation machinery of environmentally promiscuous RP4 plasmid into a minimized, synthetic construct that enables transfer of chromosomal segments between donor/recipient strains of *P. putida* KT2440 and potentially many other Gram-negative bacteria. The synthetic device features [i] a R6K suicidal plasmid backbone, [ii] a mini-Tn5 transposon vector, and [iii] the minimal set of genes necessary for active conjugation (RP4 Tra1 and Tra2 clusters) loaded as cargo in the mini-Tn5 mobile element. Upon insertion of the transposon in different genomic locations, the ability of *P. putida*-TRANS (**t**ransference of **R**P4-**a**ctivated **n**ucleotide **s**egments) donor strains to mobilize genomic stretches of DNA into neighboring bacteria was tested. To this end, a *P. putida* double mutant Δ*pyrF* (uracil auxotroph) Δ*edd* (unable to grow on glucose) was used as recipient in mating experiments, and the restoration of the *pyrF*^+^/*edd*^+^ phenotypes allowed for estimation of chromosomal transfer efficiency. Cells with the inserted transposon behaved in a manner similar to Hfr-like strains and were able to transfer up to 23% of their genome at frequencies close to 10^–6^ exconjugants per recipient cell. The hereby described TRANS device not only expands the molecular toolbox for *P. putida*, but it also enables a suite of genomic manipulations which were thus far only possible with domesticated laboratory strains and species.

Before the onset of the genomics era, *E. coli* Hfr (high frequency of recombination) strains were used to establish the first physical maps of prokaryotic chromosomes.^1^ In these strains, the F plasmid integrated in the genome provided the conjugational machinery and the origin of transfer needed to mobilize large genomic stretches toward F^®^ recipient cells. By means of interrupted conjugation experiments, a complete linkage map of different genetic markers covering the whole genome of *E. coli* was assembled. While the F sex factor was used for similar endeavors in *Salmonella typhimurium*,^2^ F-like plasmids were found to be functional only in the enterobacteria group.^3^ The discovery of new conjugative plasmids expanded those methodologies to other bacteria such as *Pseudomonas aeruginosa*(4) and *Proteus mirabilis*.^5^ Although such classical approaches have become obsolete nowadays, genome transfer assisted by conjugation has been recently applied in cutting-edge applications, such as the genome-wide codon replacement of *E. coli* driven by the hierarchical Conjugative Assembly Genome Engineering (CAGE)^6^ or the chromosome transplantation to *E. coli* minicells.^7^ Thus, continued exploitation of promiscuous conjugative plasmids represents a promising strategy for the development of similar genetic tools for other prokaryotes. Among the plethora of conjugative elements described so far, RP4 plasmid (also known as RK2, RP1, and the Birmingham plasmid) stands not only as a model of bacterial conjugation studied over the past 40 years, but also as one of the most conspicuous, broad-host range conjugative plasmids described in the literature. It mediates mating and plasmid transfer between a wide variety of Gram^–^ donors/recipients^8^ and is also capable of efficiently conjugating with Gram^+^,^9^ yeast^10,11^ and mammalian cells.^12^ Additionally, RP4 plasmid inserted in *E. coli* and *P. aeruginosa* genomes has been reported to foster some extent of genome transfer.^13,14^ RP4 is a large plasmid (60 Kb), the conjugation genes of which are split in two independent regions, Tra1 and Tra2, responsible for DNA transfer (Dtr) and the mating pair formation (Mpf) functions, respectively. The mechanism of RP4 conjugational transfer is not completely understood yet, but is known to include a concerted action of different proteins of the Tra1 Core that begins when a DNA strand transferase protein, the relaxase, recognizes the sequence of *oriT*, nicks the DNA, and covalently binds to the 5′ end. The Tra2-encoded proteins are responsible for the pili and the mating channel formation, which brings the donor and recipient cells into intimate contact. The nascent ssDNA copy, likely replicated by a rolling-circle-like mechanism, passes through the mating bridge, and the process continues until the relaxase finds the reconstituted nick site in the incoming DNA. The ssDNA is then recircularized while the complementary strand is synthesized by the recipient-encoded replication machinery.^15−20^ The versatility and broad functionality of RP4 conjugation machinery led us to adopt this system as the basis for our rational design of a genetic device capable of transforming Gram^–^ bacteria into a Hfr strain, in a manner reminiscent of the F sex factor in *E. coli*.

In this paper, yeast assembly was used to fuse Tra1 and Tra2 into a compact genetic cluster of ∼20 Kb, with the endogenous Tra1 gene regulation exerted by the *kor* genes^21^ substituted by the inducible expression system *xylS*/P_*m*_. To allow for a simple and efficient insertion of Tra1-Tra2 into the bacterial chromosome, the assembly design included a mini-Tn5 transposon (Km^R^) loaded with the Tra1-Tra2 gene cluster and also a suicidal R6K plasmid backbone. The resulting pTRANS system was tested in *Pseudomonas putida* EM42 to confirm its potential applications in nonenterobacterial strains. Two *P. putida* EM42 derivatives carrying the TRANS module in different genomic *loci* were used as donors in mating experiments. The work explained below documents the transfer of genetic markers *pyrF* and *edd* to recipient strains of *P. putida* EM42 and quantifies the frequency of transfer. DNA segments ranging from 0.16 to 1.4 Mb were transmitted to the recipients at rates between 2.6 × 10^–3^ and 3.6 ×10^–6^ trans-conjugants per recipient cell, demonstrating that *P. putida* cells acquired a Hfr-like state upon insertion of the TRANS device. The utility of the system and the potential applications in the fields of genome shuffling, combinatorial diversification and directed evolution are considerable.

## Results and Discussion

### Rationale for Designing a Synthetic Genome-Mobilizing Device

**Figure 1:**
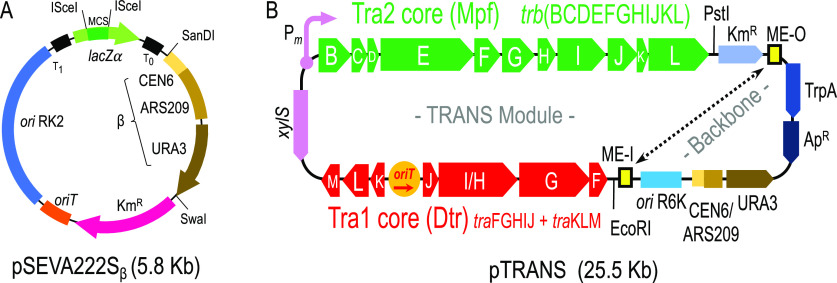
Scheme of plasmids constructed in this study. (A) Structure of the yeast shuttle vector pSEVA222S_β_: T_0_ and T_1_, transcriptional terminators; *lacZ*α-pUC19/I-SceI with the SEVA standard multicloning site (MCS) and two ISceI sites; gadget β including yeast centromeric region CEN6, the Autonomous Replication Sequence ARS209, and the URA3 gene; Km^R^, kanamycin resistance gene; *ori*T, origin of transfer; *ori* RK2, origin of replication. (B) Structure of pTRANS plasmid: *xylS*-P_*m*_, 3-methyl-benzoate inducible expression system; Tra2 Core region, gene cluster *trb*(BCDEFGHIJKL) involved in Mating Pair Formation (Mpf) functions; Km^R^, kanamycin resistance gene; Mosaic ends ME-O and ME-I, target sequences of TrpA; TrpA, hyperactive Tn5 transposase; Ap^R^, ampicillin resistance gene; CEN6/ARS209/URA3, region for partitioning, replication, and selection on *S. cerevisiae* cells; *ori* RK2, origin of replication; Tra1 Core (Dtr) showing gene clusters *tra*(FGHIJ) and *tra*(KLM) together with the origin of transfer, *oriT* (the red arrow depicts the direction of DNA transfer during conjugation). Unique sites *Eco*RI and *Pst*I are also represented.

The pTRANS plasmid was designed *ad hoc* with the purpose of cleanly inserting the conjugational machinery of RP4 into the genome of any Gram^–^ bacteria in order to generate a Hfr derivative. The complexity of this construct required the use of yeast assembly in *Saccharomyces cerevisiae* to merge all the functional modules in a single plasmid. Because a yeast replication element was mandatory in the final construct, the yeast/bacteria shuttle plasmid pSEVA222S_β_ was constructed to facilitate later construction of the pTRANS plasmid (Figure 1A): it contains three characterized SEVA modules (Ab^R^#2, Km resistance gene; *ori*#2, RK2 origin of replication; cargo#2S, *lacZ*α-pUC19/I-SceI) and a new gadget, designated as β, which includes all necessary sequences to allow replication/selection in *S. cerevisiae* yeast cells. The β gadget is located between SandI/SwaI sites of the SEVA backbone^22^ and includes the Autonomous Replication Sequence 209 (ARS209-^23,24^), the Centromer DNA 6 (CEN6-^25^) and the yeast URA3 gene.^26^ These three elements were edited to comply with SEVA rules^27^ and allow for, respectively, DNA replication, faithful segregation, and auxotrophic selection on URA^®^ yeast strains. A detailed description of plasmid construction can be found in the Supplemental Information. In this work, pSEVA222S_β_ was essentially used to amplify the β gadget for pTRANS construction.

A complete scheme of pTRANS is shown in Figure 1B, comprising several functional elements organized in a plasmid backbone (6 Kb) and the TRANS module (19.5 Kb). The plasmid backbone contains [i] a suicidal R6K origin of replication, [ii] a yeast replication/selection region CEN6-ARS209-URA (β gadget), [iii] the *bla* gene for ampicillin resistance (Ap^R^), and [iv] a modified *trpA* gene encoding a hyperactive transposase of Tn5. The TRANS module, on the other hand, is flanked by the mosaic end sequences ME-I and ME-O (targets of the TrpA transposase in mini-Tn5 transposons^28^) and includes the Tra1 and Tra2 cores of RP4 plasmid as well as a Km^R^ cassette. Tra1 core (Dtr) contains the origin of transfer (*oriT*) and two operons (*traFGHIJ* and *traKLM*). The protein complement of Tra1 is responsible for the *oriT* recognition/nicking and also mediates DNA transfer through the mating channel.^15,29,30^ On the other hand, the Tra2 Core (Mpf) consists of a gene cluster *trbABCDEFGHIJKL*, the gene products of which are involved in the mating channel and pili formation.^15,19^ These two regions encode the complete conjugation machinery of RP4 and are sufficient to promote DNA transfer through bacterial mating.^16^

The detailed assembly strategy of pTRANS is depicted in Supporting Information, Figure S1. Since the construction method relies on the homologous recombination (HR) machinery of *S. cerevisiae*, all DNA pieces must share homology with the adjacent DNA fragment of the final construct. Therefore, nine PCRs representing the functional elements of pTRANS were amplified, including overlapping regions of 0.5–0.8 Kb for adjacent fragments (i.e., PCRs 3 and 4 of Tra2). Additionally, five linkers were also constructed to provide homology between unrelated neighboring fragments. The Tra1 core, the expression of which is driven by overlapping promoters P_*traJ*_ and P_t*raK*_ (within the *oriT* sequence), was amplified from RP4 by two PCRs including the transcriptional terminators flanking the divergent relaxase (*traFGHIJ*) and leader operons (*traKLM*).^17^ The Tra2 Core was also amplified from the RP4 template by three PCRs. The design of the Tra2 Core excluded the first gene of the cluster, *trbA*, the cognate operon promoters P_*trbA*_ and P_*trbB*_ and also the global regulators *korA* and *korB*. Tra2 expression is controlled by the *trbA* repressor and also by the products of *kor* genes, which in turn are involved in the regulation of a broad number of conjugation, replication, and partitioning functions of the RP4 plasmid.^17,21,31^ Since a proficient conjugation has been reported when Tra2 cluster is expressed from a heterologous expression system,^15^ the *trbBCDEFGHIJKL* genes were placed under the control of the *xylS*-P_*m*_ expression system to elicit the endogenous regulation network. *xylS*-P_*m*_, the Km^R^ cassette, and the backbone elements (*ori* R6K, gadget β, Ap^R^ cassette, and TrpA transposase) were recruited from SEVA collection plasmids. The DNA pool composed of PCRs1–9 and Linkers 1–5 was transformed in yeast cells and, upon selection of positive yeast clones, plasmidic DNA was isolated and subsequently transformed in *E. coli*. Restriction analysis and full sequencing was performed to ensure a correct assembly and sequence (see the Experimental Section for details).

### pTRANS Activity in *E. coli*

Functionality of the RP4 minimal conjugation machinery present in the hereby described genetic tool was first tested in *E. coli* via a conjugation efficiency assay. To this end, biparental matings between *E. coli* DH5α λ*pir* (pTRANS), a donor strain sensitive to rifampicin, and *E. coli* CC118 λ*pir*, a recipient strain resistant to rifampicin, were performed. Since pTRANS confers resistance to Ap and Km, selection of trans-conjugants in Ap Km Rif media and comparison with recipient cells selected in Rif media allowed for estimation of the transfer ratio of pTRANS from the donor to the recipient strain. The Tra2 cluster was designed to be inducible by 3-methyl-benzoate (3MB), so assays were conducted in both the presence and absence of the inducer. A negative control was performed with *E. coli* DH5α λ*pir* (pBAMD1–2) + *E. coli* CC118 λ*pir*. pBAMD1-2 (Table S1) is a R6K-based plasmid with Ap^R^ and Km^R^ cassettes, an *oriT*, and also an empty mini-Tn5 transposon, thus similar to pTRANS backbone but lacking any conjugation machinery.

**Figure 2:**
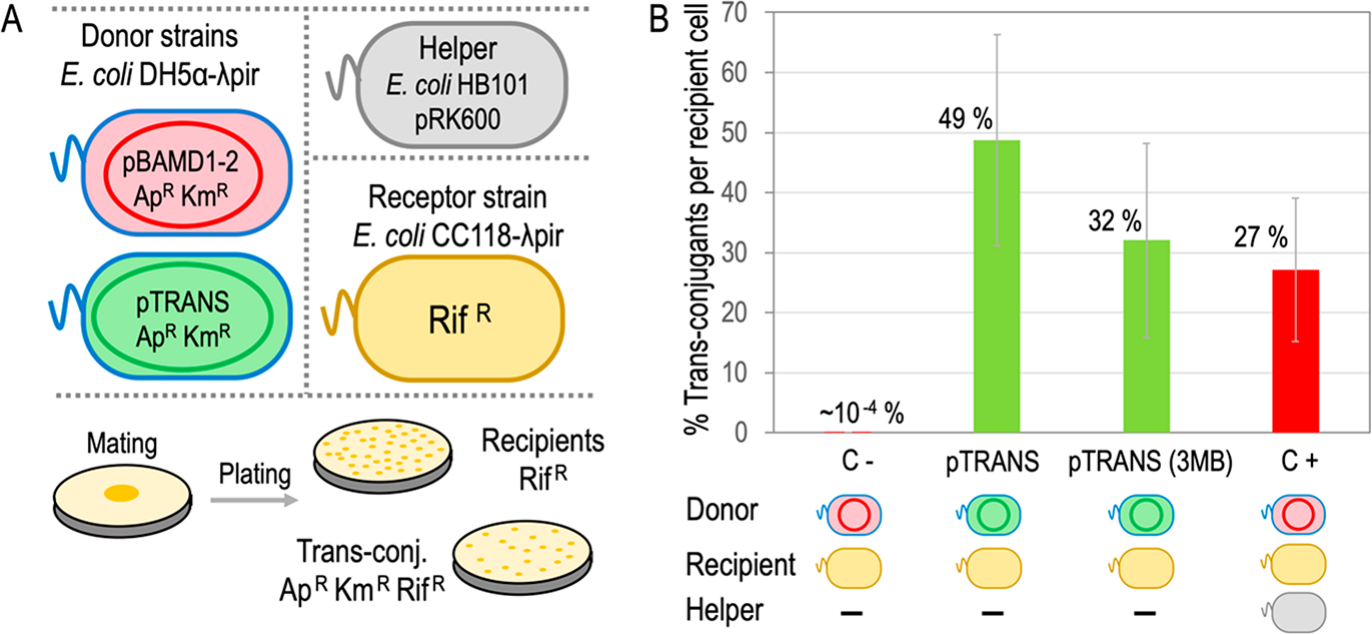
Conjugation efficiency assay. (A) *E. coli* strains used in this assay are outlined: donor bacteria DH5α harboring either pBAMD1-2 (Control) or pTRANS are Ap^R^Km^R^ (and Rif^S^), while the receptor strain *E. coli* CC118 λ*pir* is Rif resistant. Below appears an example of mating plated in selective media to quantify receptors (LB-Rif) and trans-conjugants harboring either pTRANS or pBAMB1-2 (LB-Ap Km Rif). (B) Efficiency of conjugations are represented as the percent number of trans-conjugants (enumerated as CFUs in Ap Km Rif) per recipient cell (CFUs Rif^R^). Donor and recipients used in each mating experiment are depicted below: negative control was performed with pBAMD1-2 donor while positive control was conducted in a triparental mating with the same donor and the mating helper strain *E. coli* HB101/pRK600. Conjugations with pTRANS donor were done in the presence and absence of the *xylS*-P_*m*_ inductor (3MB) during the mating procedure.

As a positive control, a similar mating with the last two strains and the helper strain *E. coli* HB101 (pRK600) was included. Results of this assays are represented in Figure 2. The negative control showed just a marginal appearance of Ap Km Rif cells (10^–4^%), probably due to spontaneous rifampicin mutants in the donor population. In contrast, 3MB-induced matings of pTRANS reached similar efficiencies to the positive control (∼30% trans-conjugants per recipient cell), demonstrating a remarkable performance of the condensed RP4 conjugation machinery present in pTRANS. Unexpectedly, experiments in the absence of 3MB yielded even higher values (∼50%), suggesting that the TRANS device worked in a constitutive fashion. While the reason for this unanticipated behavior is not clear, it is possible that an alternative promoter triggered the expression of Tra2 cluster. Since native control of the expression of the Tra2 cluster is unknown and may fail in some species, inclusion of the *xylS*/P*m* inducible system (known to function in a wide variety of Gram^–^ organisms) acts as a backup for widening the range of bacterial types that are amenable to the methodology. But this may vary with the species. Lower conjugational activity under 3MB induction could reflect in this case an excessive expression of Tra2 genes, which has been reported to greatly increase the membrane permeability to ATP, potassium, and lipophilic compounds in *E. coli* cells.^32^ Regarding the Tra1 core, P_*traK*_ seems to drive the functional expression of the *traKLM* operon.^17^ Although there is a transcriptional terminator downstream of *traM*, a read-through transcription from P_*traK*_ could explain the observed results. It is worth mentioning that a T insertion was spotted in the terminator sequence of the pTRANS construct, so involvement of this mutation in the termination performance cannot be ruled out. All in all, the results summarized in Figure 2 demonstrate that the TRANS device actively promotes conjugation in *E. coli* cells.

### Genome Transfer in *P. putida*

The TRANS sequence contains all necessary elements to promote self-mobilization from cell to cell in *E. coli*, as shown in the previous section. In the assays reported below, we interrogated the ability of the system to mobilize chromosomal regions between *P. putida* cells. To this end, specific donor and recipient strains of *P. putida* EM42 (a streamlined derivative of KT2440^33^) were constructed. As donor bacteria, a collection of strains with mini-Tn5 insertions of the TRANS module was constructed (see the Experimental Section for details).

**Figure 3:**
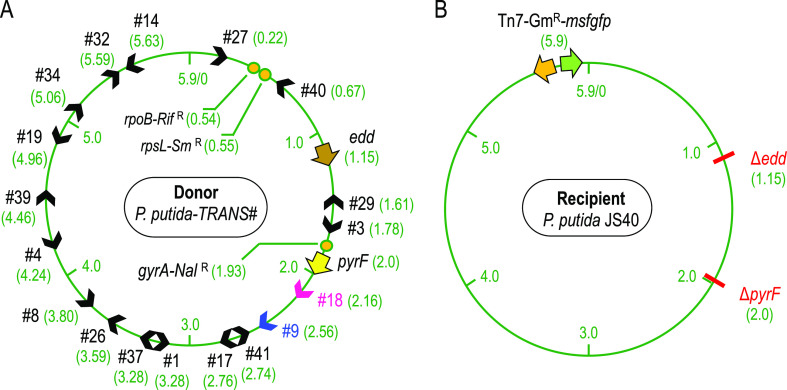
Genomic structure of *P. putida* donor and recipient strains used in this study. (A) Donor: schematic view of 18 *P. putida*-TRANS strains carrying the TRANS module in a mini-Tn5 transposon. Arrowheads represent the insertion site and the direction of DNA transfer according to *oriT* orientation within the TRANS sequence. *Loci* coordinates in Mb appears in brackets for insertions and also for the marker genes *pyrF*, and *edd*. Mutations conferring Sm, Rif, and Nal resistances are also depicted. The strains assayed in this work, *P. putida*-TRANS#9 and 18, are highlighted in blue and magenta, respectively. (B) Recipient: *P. putida* JS40 shows a double deletion Δ*pyrF* Δ*edd* and a Tn7 insertion (Gm^R^) featuring a constitutively expressed msfGFP.

Figure 3A shows 18 *P. putida*-TRANS strains for which arbitrary PCR was used to identify the genomic location of their insertion. *P. putida*-TRANS#9 and #18 clones were selected as donors, while another derivative of EM42, *P. putida* JS40, was used as the receptor strain. *P. putida* JS40 displays constitutive expression of msfGFP, resistance to Gm and a double deletion Δ*pyrF* Δ*edd* (Figure 3B). The product of the *pyrF* gene (PP_1815) is involved in uracil synthesis, so deletion mutants display uracil auxotrophy.^34^ Deletion of the *edd* gene (PP_1010), on the other hand, gives rise to mutants with impaired growth on glycolytic carbon sources since it encodes the first enzyme of the Entner-Doudoroff pathway of *P. putida*.^35,36^ Therefore, *P. putida* JS40 is an auxotroph for uracil and deficient for growth in glucose minimal media.

**Figure 4:**
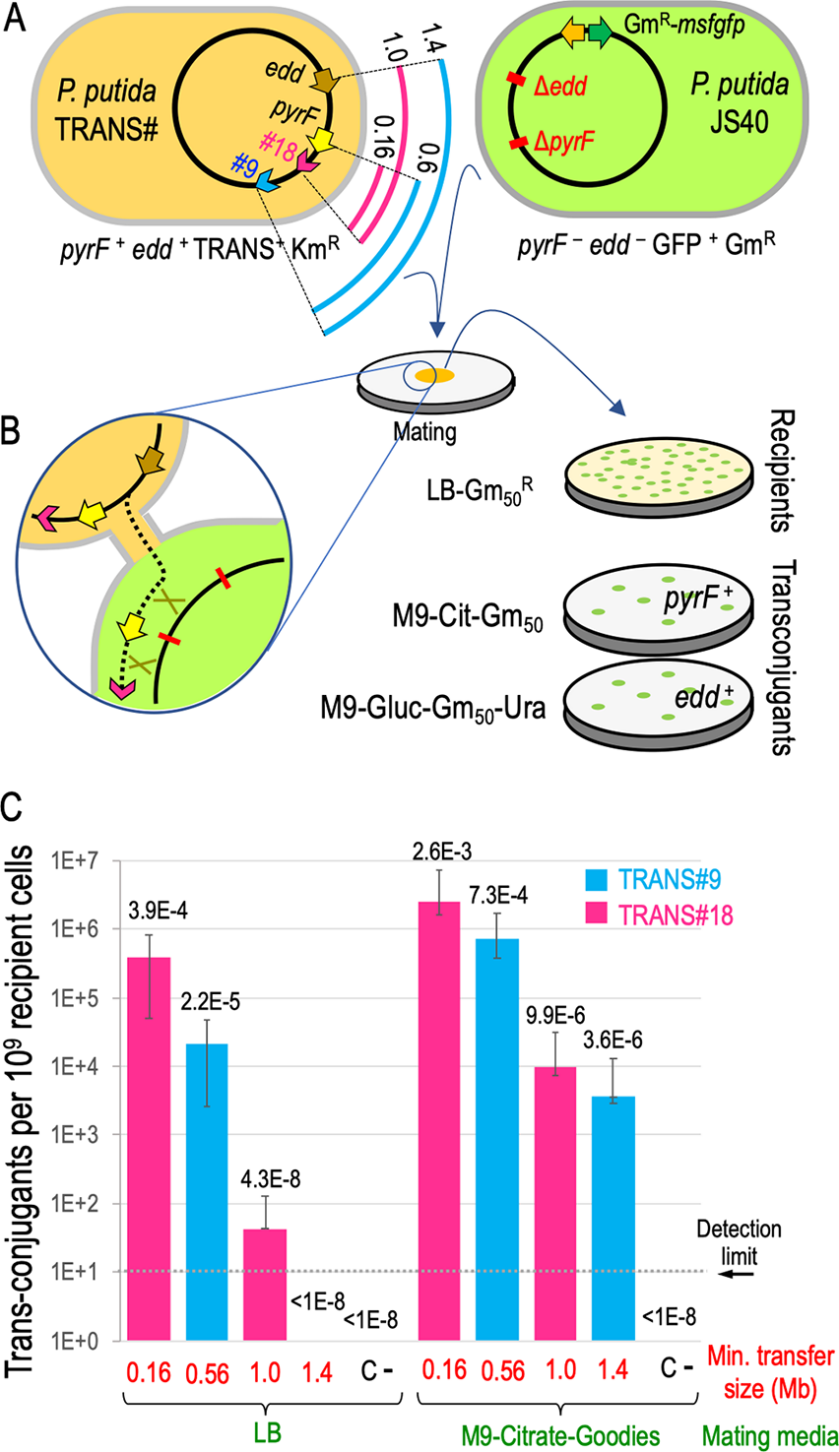
Conjugative transference of genomic segments from *P. putida-*TRANS engineered strains. (A) On the left, scheme of the two donors (*P. putida*-TRANS#) showing the length from the TRANS insertions #9 (blue arcs) and #18 (magenta arcs) to the marker genes *pyrF* and *edd* (in Mb). Arrowheads indicate the direction of genomic transfer from the *oriT*. On the right, scheme of the recipient strain *P. putida* JS40 showing the marker gene deletions and the insertion of Tn7-Gm^R^-*msfgfp*. Relevant phenotypes of depicted strains appear below. (B) Zoom-up of two mating cells sketching the conjugational transfer from *P. putida*-TRANS#18 to *P. putida* JS40. The dotted line depicts a replicating DNA strand passing through the mating bridge. Green crosses symbolize recombination events in a merodiploid recipient leading to a *pyrF*^+^ cell. Selection strategy is also shown: *pyrF* transfer is assayed in M9-citrate-Gm_50_ media (recipients able to grow without uracil), and *edd* transfer is assayed in M9-glucose-Gm_50_-Ura media (recipients growing with glucose as carbon source). (C) Efficiency of genomic transfer in two different mating media is expressed as the number of trans-conjugants per 10^9^ recipient cells. Minimal transfer size is defined as the length between a TRANS insertion (blue for #9, magenta for #18) and the marker gene assayed. C- stands for experiments with donor strain *P. putida* TA280 (lacking the TRANS device). Medians and standard deviations come from three independent replicas. Absolute frequencies (trans-conjugants per recipient cell) are also shown over the bars. The detection limit was set at 10 trans-conjugants/10^9^ recipients (1 × 10^–8^ trans-conjugants per recipient cell) since ∼10^8^ recipient cells were used routinely in these mating assays.

Figure S2 shows the phenotypic characteristics of donor and receptor strains on different selective media. On this background, the conjugational transfer of genomic stretches from donor strains to the double mutant receptor can be monitored by selection of trans-conjugants either on uracil-deficient media (*pyrF* transfer) or on media with glucose as the carbon source (*edd* transfer). Figure 4A represents the main features of the depicted strains, including the distance between the TRANS insertions #9 and #18 and the marker *loci pyrF*/*edd*. Figure 4B briefly outlines the theoretical mechanism of conjugal transfer: the replicative mobilization of the donor chromosome starting from the *oriT* of the TRANS sequence drives the nascent DNA through the cell-to-cell conjugational channel. Then, homologous recombination swaps a genomic segment into the receptor cell. Independent experiments were set up subjecting donors *P. putida*-TRANS#9 and *P. putida*-TRANS#18 to plate mating with the receptor strain *P. putida* JS40. A negative control using the same receptor and the donor *P. putida* TA280 (parental strain for donor construction lacking the TRANS module) was also done.

Trans-conjugation events restoring *pyrF*^+^ or *edd*^+^ phenotypes in the receptor strain were identified by plating the mating mixture and counting CFUs, respectively, in M9-citratre-Gm_50_ and M9-glucose-Ura-Gm_50_ selective media. Colonies from both experimental sets were further analyzed by PCR to check the integrity of the marker genes in the *P. putida* JS40 trans-conjugants (Figure S3). The total number of receptors, on the other hand, was evaluated in LB-Gm_50_. Since resistance to Gm and the presence of green fluorescence were used as double criterion to identify receptor-borne CFUs, only GFP^+^ colonies were counted as receptors in these assays.

Notice that receptor strain displays some extent of residual growth on glucose (Figure S2): this fact accounts for the observed appearance of a background of tiny colonies in M9-Glucose-Ura-Gm_50_ (data not shown). Therefore, only regular-size colonies were enumerated as *edd* trans-conjugants. It is also worth mentioning that virtually 100% of observed colonies (either in LB-Gm_50_ or in selective media) displayed a clear fluorescent signal (data not shown). With transfer efficiency defined as the number of trans-conjugants per 10^9^ receptor cells, the outcome of this exercise is represented in Figure 4C for different sizes of chromosome-mobilized regions. The minimal transfer size was defined here as the shortest DNA sequence that, once mobilized to the receptor cell, could be integrated in the *P. putida* JS40 chromosome by two or more events of HR. Therefore, this length was calculated for the genomic region spanning from the *oriT* to the assayed marker gene (*pyrF*^+^ or *edd*^+^). Results depicted in Figure 4C show that the TRANS device mediates genomic transfer between *P. putida* cells for chromosome regions ranging from 0.16 to 1.4 Megabase pairs (Mb). A first set of experiments was done developing the bacterial mating in LB media, but additional assays showed that M9-citrate-goodies mating media greatly improved transfer efficiencies. Genomic stretches of 0.16 Mb were transferred at absolute frequencies (trans-conjugants per recipient cell) of 3.9 × 10^–4^ in LB while results in M9 media reached 2.6 × 10^–3^. Larger regions of 0.56 and 1.0 Mb were mobilized at 7.3 × 10^–4^ and 9.9 × 10^–6^, respectively, in M9 matings. LB mating resulted in much lower frequencies of 2.2 × 10^–5^ (0.56 Mb) and 4.3 × 10^–8^ (1.0 Mb) trans-conjugants per recipient. The largest genomic region assayed (1.4 Mb) was mobilized from TRANS#9 donor using *edd* as the marker gene, accounting for an absolute transfer frequency of 3.6 × 10^–6^ in M9 media. In the case of LB mating media, no trans-conjugants could be observed, probably because the frequency of transfer fell below the assay detection limit (∼1 × 10^–8^). The negative control showed no trans-conjugant CFUs. The reason behind the dependence of performance efficiency upon mating media (with a differential transfer rate higher than 10-fold) is unclear, but could be due to the positive effect of trace elements (so-called *goodies* in the media composition) on the conjugation and DNA transfer process.

In any case, the presented results attest that regions accounting for almost 25% of *P. putida* genome (1.4 out of 6.0 Mb) can be successfully transferred to a recipient strain by TRANS-mediated conjugation. In *E. coli*, genomic transfer mediated by the F episome during CAGE assembly^6^ yielded frequencies of 1 × 10^–4^ (0.15 Mb) and 2.5 × 10^–6^ (half *E. coli* genome-2.3 Mb). F plasmid integrated in the *E. coli* genome has been shown to produce Hfr strains with frequencies of transfer close to 1 × 10^–1^,^37^ while *E. coli* laboratory strains with RP4::Mu integrations (i.e., S17–1) transfer genomic segments at 1 × 10^–4^.^13^ However, few works report genome mobilization by conjugation out of *E. coli*: in *P. aeruginosa* the conjugative plasmids FP2 and RP1 (=RP4), after spontaneous integration in the genome, generated Hfr-like strains able to mobilize genomic regions at frequencies around 1 × 10^–3^ (early markers) and 2 × 10^–8^ (late markers) recombinants per donor cell.^14,38,39^ In contrast, the tool hereby described expands very significantly the range of species that can be set for massive chromosomal exchanges.

## Conclusion

To the best of our knowledge, this is the first time that genome transfer has been programmed and verified in *P. putida*, demonstrating the potential of the TRANS module to generate Hfr-like strains in environmental bacteria. Given the high promiscuity of the RP4 conjugation machinery, the same strategy can be easily applied to other Gram^–^ strains and species of interest. Unlike the use of chromosomal integrations of complete conjugative plasmids, which are difficult to obtain and show limited insertion sites in bacterial genomes, pTRANS offers the possibility of a rapid, efficient and unbiased delivery of the conjugation module along with the bacterial chromosome at stake. It cannot escape one’s notice that the ease of intraspecies and interspecies genome mobilization enabled by pTRANS opens a wide array of applications that were thus far limited to *E. coli* and very closely related species. Chromosomal shuffling^40−42^ between otherwise distant genomes appears to be a particularly appealing outlook, as it may allow combinations of desirable traits that are originally present in separate hosts.^43^ Furthermore, the hereby reported efficiencies of *P. putida*-Hfr strains, even though sufficient for many practical applications, could be improved in various ways, for example, fine-tuning of the Tra machinery expression and mutagenesis of the Tra cores^44^ and coexpression of factors enhancing recombination/ssDNA protection. We thus argue that the genetic tool hereby documented and its possible spinoffs will make possible an unprecedented range of genetic manipulations with nonmodel environmental bacteria.

## Experimental Section

### Conjugation Efficiency Assay

The ability of pTRANS to mediate autoconjugative transfer was assayed by mating *E. coli* DH5α λ*pir* (Rif ^S^) as the donor bacteria and *E. coli* CC118 λ*pir* (Rif^R^) as the recipient bacteria. Both strains encode the λ*pir* replication protein in the chromosome and support plasmids with R6K origins of replication, but differ in Rifampicin sensitivity. *E. coli* DH5α λ*pir* was first transformed with pTRANS (R6K, Ap^R^Km^R^), and a pBAMD1–2 (R6K, Ap^R^Km^R^) bearing strain was also constructed for positive/negative controls. Independent mating experiments were set up with recipient *E. coli* CC118λ*pir* plus the donors *E. coli* DH5α λ*pir* (pTRANS) and *E. coli* DH5α λ*pir* (pBAMD1-2) as negative control. A positive control for conjugation was also included with a triparental mating containing *E. coli* DH5α λ*pir* (pBAMD1-2), *E. coli* CC118 λ*pir*, and the mating helper strain *E. coli* HB101 (pRK600). Bacterial strains were grown overnight in 3 mL of LB supplemented with appropriate antibiotics: Ap Km for *E. coli* DH5α λ*pir* bearing either pTRANS or pBAMD1-2, *Cm* for *E. coli* HB101 (pRK600) and Rif for *E. coli* CC118 λ*pir*. One milliliter of each culture was centrifuged at 11000 rpm/1 min and resuspended in 1 mL of 10 mM MgSO_4_. The OD_600_ of the resuspended samples was measured and adjusted to 1.2 with the same media. Individual experiments were set up mixing 100 μL of each strain into an Eppendorf tube. One milliliter of 10 mM MgSO_4_ was added, the sample was vortexed briefly, and it was centrifuged 1 min at 11000 rpm. After supernatant removal, the cellular pellet was resuspended in 10 μL of 10 mM MgSO_4_ by gentle pipetting. The 10–14 μL drop was placed on top of a LB-agar plate, air-dried for 10 min, and incubated 18 h at 37 °C in an upward position. For the mating of *E. coli* DH5α λ*pir* (pTRANS) + *E. coli* CC118 λ*pir*, both noninduced and induced experiments were performed using, respectively, LB-agar and LB-agar supplemented with 3-methyl benzoate (3MB) 1 mM. After incubation, bacterial patches were scraped out with an inoculation loop and resuspended in 1 mL of 10 mM MgSO_4_. Serial dilutions were prepared in the same media and plated in LB agar plates supplemented with Rif and Rif-Ap-Km. Plates were incubated 24 h at 37 °C, and colony counts were taken. Conjugation efficiency was calculated as the ratio of trans-conjugants (Ap Km Rif resistant colonies) per recipient cell (Rif^R^ colonies). Three independent replicas were performed for each experiment and the media and standard deviations were represented graphically in percentages.

### Genome Transfer Assays in *P. putida*

The ability of *P. putida* TRANS#9 and *P. putida* TRANS#18 donors (Figure 3A) to transfer genome determinants by conjugation was assayed in biparental matings between each donor and the recipient *P. putida* JS40 (Figure 3B). A negative control with donor *P. putida* TA280 (the ancestral strain of TRANS variants, devoid of conjugation machinery in the genome) was also included. Bacterial strains were grown overnight in 3 mL of LB supplemented with appropriate antibiotics (Km for *P. putida* TRANS# strains, Gm_50_ for *P. putida* JS40, Sm_100_ for *P. putida* TA280). Cultures were diluted to OD_600_ 0.1 in 20 mL of fresh LB with the same antibiotics and incubated in 150 mL Erlenmeyer flasks (30 °C/170 rpm) until they reached the exponential phase (OD_600_0.4–0.6). A volume of culture accounting for 0.5 units of OD_600_ (i.e., 1.25 mL of a sample OD_600_ = 0.4) was centrifuged at 11000 rpm/1 min and resuspended by gentle pipetting in 0.5 mL of washing solution (either 10 mM MgSO_4_ or M9-citrate-goodies, depending on the mating media assayed; see below for more details). A 0.5 mL sampling of each donor and recipient strains was pooled together in a 15 mL Eppendorf tube, briefly mixed by vortex, and centrifuged for 1 min at 11000 rpm. Supernatant was removed carefully and the pellet was resuspended in 10 μL of washing solution. The 10–14 μL drop was placed on top of an agar plate. Two different agar media were assayed for matings: LB-agar (washing media used was 10 mM MgSO4) and M9-Citrate-Goodies-agar (washing media: liquid M9-Citrate-Goodies). Samples were air-dried for 10 min and incubated 18 h at 30 °C in upward position. After incubation, the bacterial patch was recovered using a sterile inoculation loop, resuspended in 1 mL of the appropriate washing media and serial dilutions were performed in the same media. In general, dilutions 10^–4^–10^–6^ were plated in LB-Gm_50_ agar, while 10^–1^–10^–3^ dilutions were plated in M9-Citrate-Gm_50_ (selection of *pyrF*^+^ recipients) and M9-Glucose-Ura-Gm_50_ (selection of *edd*^*+*^ recipients). High Gm concentrations (50 μg/mL) were used to minimize the occurrence of spontaneous Gm resistant donors. Plates were incubated 48 h at 30 °C, and CFUs showing green fluorescence were counted. Genome transfer efficiency was calculated as the ratio of trans-conjugants (Gm_50_^R^-GFP^+^-*pyrF*^+^ or Gm_50_^R^-GFP^+^-*edd*^+^ colonies) per recipient cell (Gm_50_^R^ colonies). The ratios were then normalized to 10^9^ recipients. Three independent replicas were performed for each experiment and the media and standard deviations were represented graphically (Figure 4C). Twenty selected colonies from both types of trans-conjugants were further analyzed by PCR to check the presence of intact genes *pyrF* (oligos pyrF-F/pyrF-R; *T*m, 52 °C; Te, 1 min) or *edd* (oligos edd-check-F/edd-check-R; Tm, 55 °C; Te, 1.5 min). Correct amplicon size (1.2 Kb for *pyrF* and 1.5 Kb for *edd*) was found in all trans-conjugants tested and also in the donor strain, while the receptor strain showed the expected size for *pyrF* and *edd* deletions (0.5 Kb in both cases) (Figure S3).

## Supporting Information Available

Strains and media; general procedures and primers; construction of *P. putida* strains for genome transfer assays; yeast assembly methods and plasmid construction; assembly of pSEVA222S_β_; assembly of pTRANS; figures and tables supporting the text (PDF)
