# Supplementary material for: Refactoring the Conjugation Machinery of Promiscuous Plasmid RP4 into a Device for Conversion of Gram-Negative Isolates to Hfr Strains
Source: ACS Synth Biol. 2021 Mar 22;10(4):690–7. doi: 10.1021/acssynbio.0c00611 (PMC8483437; doi:10.1021/acssynbio.0c00611)
Supplement: Supplementary file 1 — sb0c00611_si_001.pdf [file sb0c00611_si_001.pdf]

SUPPORTING INFORMATION

Refactoring the conjugation machinery of promiscuous plasmid RP4 into a device for conversion of Gram-negative isolates to Hfr strains

by

Jillian Silbert, Victor de Lorenzo and Tomás Aparicio

Systems and Synthetic Biology Program, Centro Nacional de Biotecnología (CNB-CSIC), Campus de Cantoblanco, Madrid 28049, Spain.

SUPPLEMENTAL METHODS

Strains and media. The strains of *S. cerevisiae*, *P. putida and E. coli* employed in this work are listed in Supplementary Table S1. *S. cerevisiae* was routinely grown at 30 ºC/shaking (170 rpm) in liquid YPD (20 g L^{-1} Peptone, 10 g L^{-1} Yeast Extract and 20 g L^{-1} dextrose) or SC-Ura (6.7 g L^{-1} Yeast Nitrogen Base w/o amino acids, 1.92 g L^{-1} Yeast Synthetic Drop-out medium Supplement without uracil and 20 g L^{-1} dextrose). For agar plates, 2% w/v of agar was added to liquid media and autoclaved. Yeast stocks were made in liquid YPD with 15% glycerol and stored at -80 ºC. *E. coli* and *P. putida* strains were grown in liquid LB [1] with shaking (170 rpm) at 37 ºC and 30 ºC, respectively. M9 minimal media was prepared according to[1] and supplemented with 0.2% (w/v) citrate or glucose as carbon sources and 20 µg ml^{-1} of Uracil (Ura) when stated. 2.5 ml of Goodies Solution (300 mg L^{-1} HBO_3, 50 mg L^{-1} $ZnCl_2$, 30 mg L^{-1} $MnCl_2•4H_20$, 200 mg L^{-1} $CoCl_2$, 10 mg L^{-1} $CuCl_2•2H_20$, 20 mg L^{-1} $NiCl_2•6H_20$, 30 mg L^{-1} $NaMoO_4•2H_20$, 2.5 g L^{-1} FeSO4•$7H_20$, and 61.62 g L^{-1} MgSO4•$7H_20$) were added per liter of M9-Citrate agar media when necessary. Antibiotics were included in the growth media at the following concentrations: 150 µg ml^{-1} of ampicillin (Ap), 50 µg ml^{-1} of kanamycin (Km), 30 µg ml^{-1} of chloramphenicol (Cm), 100 µg ml^{-1} of streptomycin (Sm), 100 µg ml^{-1} of rifampicin (Rif) and 50 µg ml^{-1} of nalidixic acid (Nal). Gentamicin (Gm) was added at 10 µg ml^{-1} for *E. coli* and 15 µg ml^{-1} for *P. putida* growth in liquid media. Gm 50 µg ml^{-1} was used for selection of *P. putida* trans-conjugants in solid media during genome transfer experiments.

General procedures and primers. DNA manipulations were carried out following standard protocols [1] and according to manufacturer recommendations. Primers used in this study are listed in Table S3 and were purchased from Sigma-Aldrich (St. Louis, USA). Standard plasmid purifications were performed with the QIAprep® Spin Miniprep Kit (Qiagen, Valencia, USA). In the case of RP4, plasmid isolation kits worked very poorly for this large plasmid. Therefore, DNeasy® UltraClean® Microbial Kit (Qiagen) was used to isolate the whole DNA content of *E. coli* cells hosting RP4 and DNA samples were used as plasmid template for PCR reactions. In all cases, plasmid samples used as PCR templates were isolated from 5 ml culture and diluted 1:100 prior to amplification. DNA amplifications aimed for plasmid construction were performed with Q5 polymerase (New England Biolabs, Ipswich, USA). Diagnosis PCRs were conducted with DNA Amplitools Master Mix (Biotools, Madrid, Spain) and amplicon purifications were done with the

Nucleospin® Gel and PCR Clean-up Kit (Macherey-Nagel, Düren, Germany). Localization of mini-Tn5 insertions was performed via the arbitrary PCR amplification protocol outlined by [2]. DNA sequencing was outsourced to Macrogen (Spain). Special protocols were used for plasmid purification and colony PCR in *S. cerevisiae* (see "Yeast Assembly Methods" below). Transformation of *E. coli* laboratory strains was carried out with chemically competent cells using the $CaCl_2$ method [1]. *P. putida* was transformed with plasmids via tripartite mating as described in [3] and selected in solid M9 minimal media supplemented with 0.2% w/v citrate and appropriate antibiotics. Other mating protocols used in this work are explained elsewhere in the *Experimental Procedures* Section.

Construction of *P. putida* strains for Genome transfer assays. Donor strains *P. putida*-TRANS#X are derivatives of *P. putida* EM42 [4] with the TRANS device inserted in the genome via a mini-Tn*5* transposon. They also display three mutations conferring Sm, Rif and Nal resistance and were constructed as follows: *P. putida* EM42 harboring the plasmid pSEVA2514-*rec2* was first subjected to one cycle of recombineering with mutagenic oligos SR, RR and NR using the procedure outlined in [5]. Mutations introduced by these oligos in target genes *rpsL* (PP_0449), *rpoB* (PP_0447) and *gyrA* (PP_1767) are translated into resistance to Sm, Rif, and Nal, respectively (see Table S3 for details). After selection on LB-agar plates supplemented with the three antibiotics, triple resistant colonies were streaked in the same media and the target genes were amplified with oligo pairs rpsL-Fw/rpsL-Rv, rpoB-F/rpoB-R, and gyrA-F/gyrA-R. The correct sequence changes were verified by sequencing the amplicons. One selected clone was cured of pSEVA2514-*rec2* by iterative cultivation in LB without Km, obtaining the strain *P. putida* TA280. In order to insert the TRANS device into this strain, a bi-parental mating was performed as follows: *P. putida* TA280 and *E. coli* TransforMax™ EC100D™ *pir* + (pTRANS) were inoculated, respectively, in 3 ml LB-Sm and 3 ml LB-ApKm. After overnight growth, cultures were adjusted to OD_{600} ~1.0 and 1 ml of each was centrifuged 1 min/11000 rpm and resuspended in 1 ml of 10 mM MgSO4. Aliquots of 200 μl were mixed in an Eppendorf tube, 1 ml of 10 mM MgSO4 was added, and the aliquots were then spun down as before and resuspended in 10 μl of the same solution. This sample was placed on top of a LB-agar plate and incubated 18 h at 30 ºC. The cellular patch was scraped out with an inoculation loop and resuspended in 1 ml of 10 mM MgSO4. The bacterial suspension was plated in M9-Citrate-Km agar plates to select the *P. putida* TA280 trans-conjugants with mini-Tn5 insertions in

the genome. After overnight incubation at 30 ºC, around 200 colonies were obtained. Mini-Tn5 localization of 32 clones was performed as described in [2] by two rounds of arbitrary PCRs with oligo pairs MEO-Km-extF/ARB6 and MEO-Km-intF/ARB2. Eighteen representative clones of *P. putida*-TRANS#X with insertions throughout the genome were selected (Fig. 3) and two of them (#9 and #18) were assayed in this work.

The recipient strain was constructed by tagging *P. putida* EM42 Δ*pyrF*Δ*edd* [6] with msfGFP via a modification of the delivery system described by [7]. Briefly: a tetra-parental mating was set up with receptor *P. putida* EM42 Δ*pyrF*Δ*edd*, donor *E. coli* (pTn7-M-PEM7-GFP) and the helper strains *E. coli* HB101 (pRK600) -for conjugation- and *E. coli* (pTNS2) -for transposition. The four strains were grown overnight in 3 ml LB supplemented with appropriate antibiotics (Table S1) and mated applying the protocol explained in the previous paragraph. Selection was made in M9-Citrate-Gm-Ura solid media and after incubation at 30 ºC for 18 hours, colonies showed green fluorescence. Some of them were streaked in the same media and subjected to diagnostic PCRs (primer pairs PS2/PP5408-F and PEM7-F/Tn7-GlmS) to confirm the correct integration of the transposon in the *att*Tn7 *locus*. PCR analysis yielded the expected bands of 2.2 Kb and 1.2 Kb, respectively (data not shown). The double deletion Δ*pyrF* Δ*edd* makes the obtained *P. putida* JS40 both uracil auxotrophic and unable to grow on media utilizing glucose as the sole carbon source, while the Tn7 insertion confers constitutive fluorescence and resistance to Gm. To properly check such a phenotype, this strain and donor *P. putida*-TRANS#9 were grown in different culture media (Fig. S2).

Yeast assembly methods and plasmid construction. The procedure for assembling plasmid constructs via homologous recombination in the yeast *Saccharomyces cerevisiae* followed the pipeline described in [8] with several modifications. In short, PCR amplicons were designed as contiguous DNA fragments with overlapping regions. The overlaps ranged from 40 nucleotides to 0.8 Kp in length depending on the construct and were generated [i] as complementary tails included in the PCR primers [ii] as part of the PCR amplicons for adjacent sequences of a long DNA unit or [iii] as PCR linkers produced *ad-hoc* for non-adjacent fragments (see details below). DNA fragments designed for a given plasmid construct were PCR amplified and the products were purified and quantified. Equimolar mixtures of PCR fragments were used for yeast transformation

(see individual assemblies for details). Yeast were transformed via a modified version of the lithium acetate [9]: 2 mL YPD were inoculated with *S. cerevisiae* CRY1-2 and incubated overnight at 30 ºC with shaking. The next day, 25 ml of fresh YPD were inoculated with 1 ml of the overnight culture and grown at 30 ºC/170 rpm. Culture was monitored until OD_{600} reached ~1.0 (around 5 hours). The cells were then spun at 3000 rpm for 5 minutes, then washed with 20 ml water, centrifuged in the same conditions and washed again with 20 ml of freshly made TE/LiOAc solution (100 mM lithium acetate at pH 7.5, 10 mM Tris-HCl, 1 mM EDTA). After centrifugation, cells were resuspended in 0.25 ml TE/LiOAc solution. 50 µl were moved to a new Eppendorf tube and spun briefly (3000 rpm/ 10 seconds) before removing the supernatant. The following solutions were added in the subsequent order: 240 µl 50% PEG-3350, 72 µl TE/LiOAc, 5 µl of denatured Herring sperm DNA (Promega D1811, 10 mg/ml; sample denatured upon arrival at 95 ºC/ 5 min, then incubated 10 min in ice, aliquoted and stored at -20 ºC until use) and the desired amount of DNA to be transformed. When necessary, the DNA sample pool was concentrated in a SpeedVac. The final volume was adjusted to 360 µl by addition of water and the transformation mixture was carefully resuspended by pipetting up and down several times. The mixture was incubated at 30˚C for 30 minutes with gentle agitation every 10 minutes to avoid cell sedimentation. Then, heat shock at 42 ˚C was applied for another 30 minutes with gentle shaking every 10 min. Afterwards, the cells were spun for 10 seconds at 4000 rpm and resuspended in 1 ml TE solution (10 mM Tris-HCl at pH 7.5, 1 mM EDTA) before being plated onto SC-Ura selection plates. Approximately 48 hours of incubation time at 30 ºC was necessary for appropriate growth of colonies. Colonies were then streaked onto SC-Ura plates and yeast colony PCRs were conducted to confirm successful transformations. For that purpose, each colony was resuspended in 50 µl of Solution 1 (Zymoprep™ Yeast Plasmid Miniprep II kit- ZymoResearch, Irvine, USA) and zymolase (REF #E1004, ZymoResearch, Irvine, USA) was added to a final concentration of 20 U/ml. The mixture was then incubated at room temperature for 10 minutes before centrifugation (10 seconds/ 4000 rpm) and elimination of the supernatant. The cell pellet was heated at 95˚C for 5 minutes and then resuspended in 50 µl H_2O. Five µl of the suspension were used as template in a standard PCR reaction of 25 µl total volume. After gel visualization, a yeast miniprep was conducted for positive clones of *S. cerevisiae*. Yeast streaks were inoculated in 2 ml of SC-Ura liquid media and grown overnight. The protocol outlined in the Zymoprep™ Yeast Plasmid Miniprep II kit (ZymoResearch, Irvine, USA) was applied to 200 µl of the overnight cultures to isolate plasmid DNA. After elution

step, plasmid samples were evaporated in SpeedVac to concentrate them into a final volume of 10 µl. Five µL of this plasmid sample were then transformed into *E. coli* competent cells. Cells were plated in LB-Agar supplemented with appropriate antibiotics and resulting colonies were checked by miniprep, restriction analysis and sequencing (details of individual assemblies can be found in the following sections). More detailed information on buffer/reagents preparation can be found in [8].

Assembly of pSEVA222S$_{\beta}$ (GenBank MW119277). pSEVA222S$_{\beta}$ is a canonical SEVA plasmid featuring a RK2 origin of replication, a Km^{R} cassette, a cargo *lacZ*α-pUC19/I-SceI and a new gadget (β) which allows replication and selection in *S. cerevisiae* cells. The first three elements were PCR amplified from SEVA plasmids (see below) and the β gadget was recruited from plasmid pRS416. It contains the ARS209 origin of replication, the CEN6 centromeric sequence and the URA gene. A minimized version of each single element was designed to comply with the SEVA rules [10], removing non-functional DNA stretches from the original template. By the same token, the design removed prohibited restriction sites SwaI and PstI from the cognate sequence of CEN6 and URA, respectively. After amplification, purification and quantification of PCR-A (*lacZ*α-pUC19/I-SceI from pSEVA212S), PCR-B (CEN6/ARS209 from pRS416, SwaI curated), PCR-C (partial sequence of URA3 from pRS416), PCR-D (partial sequence of URA3 from pRS416), PCR-E (sewing PCR of PCR-C+PCR-D to amplify the complete URA3 gene cured of a PstI site) and PCR-F (*ori* RK2 and Km^{R} cassette from pSEVA2214), ~ 20 fentomols of the fragments PCR-A, -B, -E and -F were mixed and transformed into *S. cerevisiae* CRY1-2 following the procedure described above. In order to allow the assembly in *S. cerevisiae* via homologous recombination, amplification primers were added with sequence tails aimed to generate homologous regions of 40 nt between adjacent fragments. Details of PCR amplifications, templates, oligonucleotides and size/ functions of each amplified unit are depicted in Tables S2 and S3. After selection and re-streaking on SC-Ura agar plates, plasmid DNA from two selected *S. cerevisiae* clones was purified and electroporated into *E. coli* CC118 competent cells. Samples were plated on LB-Km agar and one colony from each transformation was first analyzed by miniprep and restriction analysis, then fully sequenced to ensure the accuracy of the construct. pSEVA222S$_{\beta}$ can replicate both in *S. cerevisiae* and *E. coli*. Replication in *P. putida* KT2440 was also verified (data not shown). Being endowed with a RK2 broad-host-range origin of replication, pSEVA222S$_{\beta}$ can potentially act as a

shuttle vector between any Gram⁻ bacteria and *S. cerevisiae* and work as a Yeast Capture Vector for recruitment of large genomic fragments.

Assembly of pTRANS (GenBank MW119276). A scheme of the design and assembly appears in Supplementary Fig. S1. Details of PCR amplifications, templates, oligonucleotides and size/ functions of each amplified unit are depicted in Supplementary Tables S2 and S3. The set of 9 structural PCRs and 5 linker PCRs used in this assembly were obtained as follows: five PCR fragments (~ 4 kb-long each) were produced to amplify the Tra1 and Tra2 regions of plasmid RP4. The two fragments representing Tra1 (PCR-1 and -2) were designed to have an overlapping region of 0.8 Kb. The three fragments from Tra2 (PCR-3, -4 and -5) were designed such that the PCR-4 overlapped with the other two fragments by 0.5 Kb and 0.8 Kb, respectively. Additional two PCR fragments were amplified from pBAMD1-2 to obtain a plasmid backbone devoid of the *oriT*: PCR-6 provided the R6K origin of replication, the ME-I edge of the Tn5 cassette and a part of the MCS (Multi Cloning Site) ending in the EcoR-I site; while the PCR-7 provided the *trpA* transposase gene, the Ap^R and Km^R resistance genes, the ME-O edge of the Tn5 cassette and part of the MCS ending in the Pst-I site. The *xylS*-P_m promoter system was obtained from pSEVA258 (PCR-8). An additional fragment, consisting of genes necessary for plasmid replication (CEN6/ARS209) and selection (URA) within *S. cerevisiae*, was amplified from pSEVA222S_β (PCR-9). Finally, 5 linker PCRs were produced to provide bridges of homology between the plasmid structural regions that would neighbor each other in the final design. Every linker was constructed via two PCR reactions of ~ 0.5 Kb each (hemi-linkers). A ~ 20 bp tail of homology was included in one of the primers, allowing the fusion of both hemi-linkers into a single molecule of ~ 1.0 Kb by sewing PCR. The 14 DNA fragments (PCR-1 to 9 and Linker-1 to 5) were transformed in *S. cerevisiae* CRY1-2 and plasmid DNA from two selected clones was electroporated into *E. coli* TransforMax™ EC100D™ *pir*$^+$ electrocompetent cells (Cat. Nº ECP09500- Lucigen, Middleton, USA) following manufacturer recommendations. This strain provides the *pir* gene in *trans*, allowing the propagation of plasmids with a R6K origin of replication. Selection was performed in LB-Ap Km agar plates, and one colony from each transformation was checked by miniprep and restriction analysis. Both clones were fully sequenced to ensure the accuracy of the construct. The resulting DNA diverged from the published sequences in 4 bases i.e. a T insertion in position 7611 (potential bi-directional terminator in Tra1 Core, downstream the gene *traM*), a T$\rightarrow$ C change in position 8906 (intergenic region in *xylS*-P_m),

a G$\rightarrow$ A change at site 15821 (Tra2 Core, $Gly_{67}Asp$ change in *trbH*) and a G$\rightarrow$ A change at site 17095 (Tra2 Core, silent mutation in Gln_{329} of *trbI*). These changes had no apparent effect in the activity of the genetic device. It is worth mentioning that the design was such that all necessary recombination events had DNA substrates with homology arms of 0.5-0.8 Kb to facilitate the assembly procedure in *S. cerevisiae* cells. This design included a sewing PCR between PCR-6 (*ori* R6K) and PCR9 (CEN6/ARS209-URA), allowed by the tail of homology (20 bp) included in the primer oriR6K-F. Therefore, the generated fragment PCR-6+9 could be connected with PCR-1 (Tra1-1) by Linker-1. However, this sewing PCR could not be obtained. The assembly of pTRANS with the separated fragments PCR-6 and PCR-9 was successful just relying on the short homology included in PCR-6 (denoted by a blue asterisk in Supplementary Fig. S1)

SUPPLEMENTAL FIGURES

Figure S1. Detailed strategy of pTRANS construction.

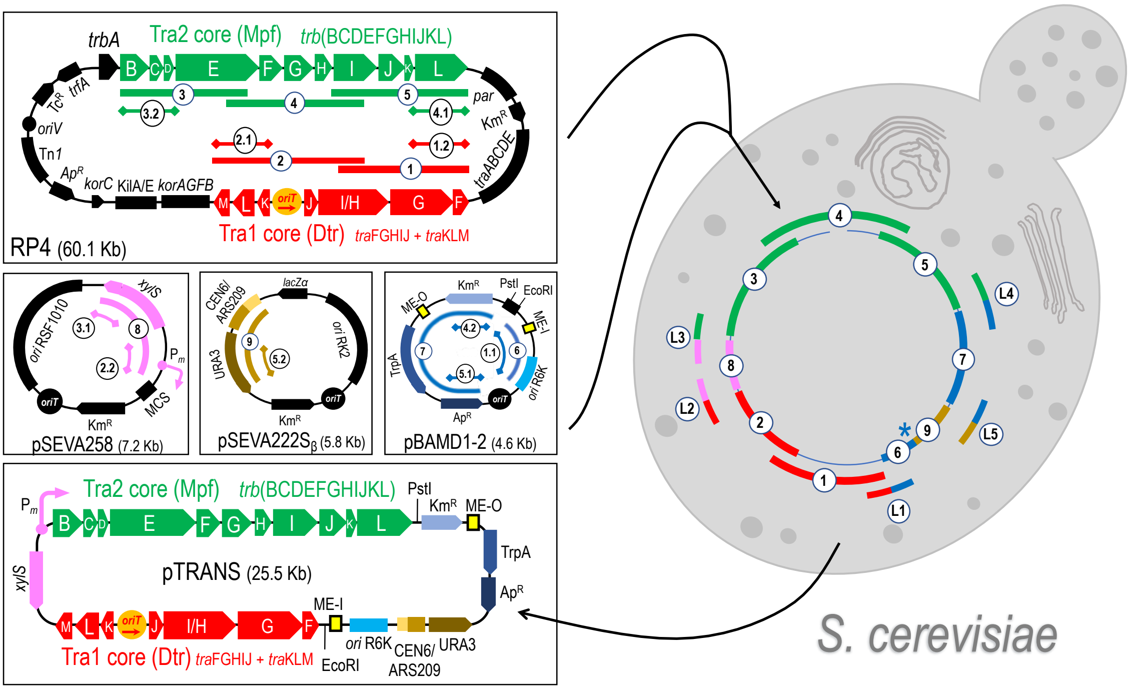

The left panels show the plasmid templates used for amplifications. Inside each plasmid, straight and stroked bars represent, respectively, structural and hemi-linker PCRs. Numbers in circles refer to PCR/hemi-linker annotation found in Table S2. On the right, the assembly scheme of pTRANS via homologous recombination is shown inside a *S. cerevisiae* cell, with the structural PCRs in the inner circle and the linker PCRs in the external circle. All recombination events were provided with homology regions of 0.5-0.8 Kb, with the exception PCR6/PCR9 connection: the blue asterisk indicates the short homology region of 20 bp included in the primer oriR6K-F of PCR6. The pTRANS final construct is also shown in the left-bottom panel.

Figure S2. Phenotypic characterization of donor and recipient strains used in this study.

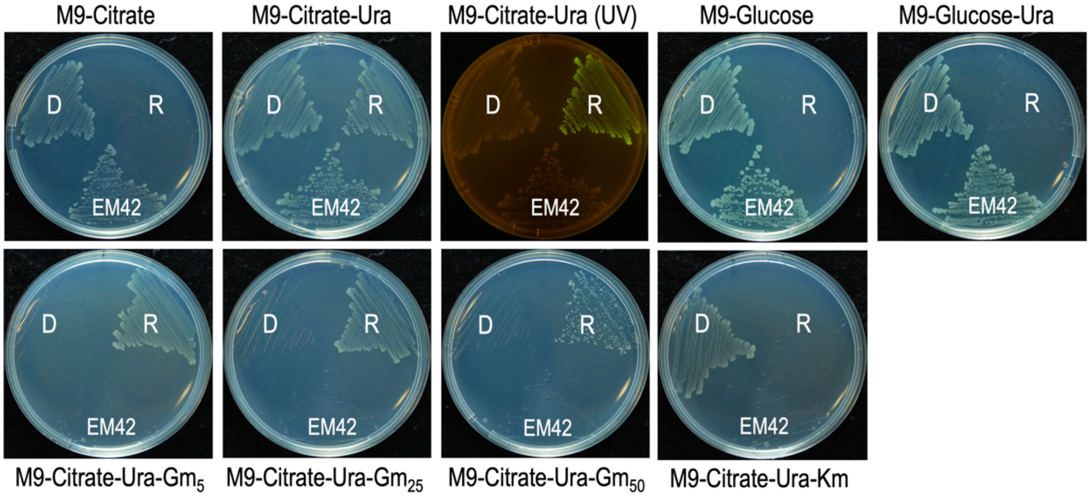

Growing phenotypes of *P. putida*-TRANS#9 (donor, D), *P. putida* JS40 (receptor, R) and *P. putida* EM42 ancestral strain were assayed in different M9 solid media. Regardless the addition of uracil, donor strain grew both in citrate and glucose carbon sources, showing Km resistance and Gm sensitivity. Receptor strain was Km sensitive, had uracil-dependant growth and showed only residual growth on glucose carbon source, supporting Gm concentrations up to 50 μg/ml. Green fluorescence of *P. putida* JS40 can be seen in M9-Citrate-Ura under UV light.

Figure S3. PCR analysis of *pyrF* and *edd* genes in *P. putida* JS40 trans-conjugants.

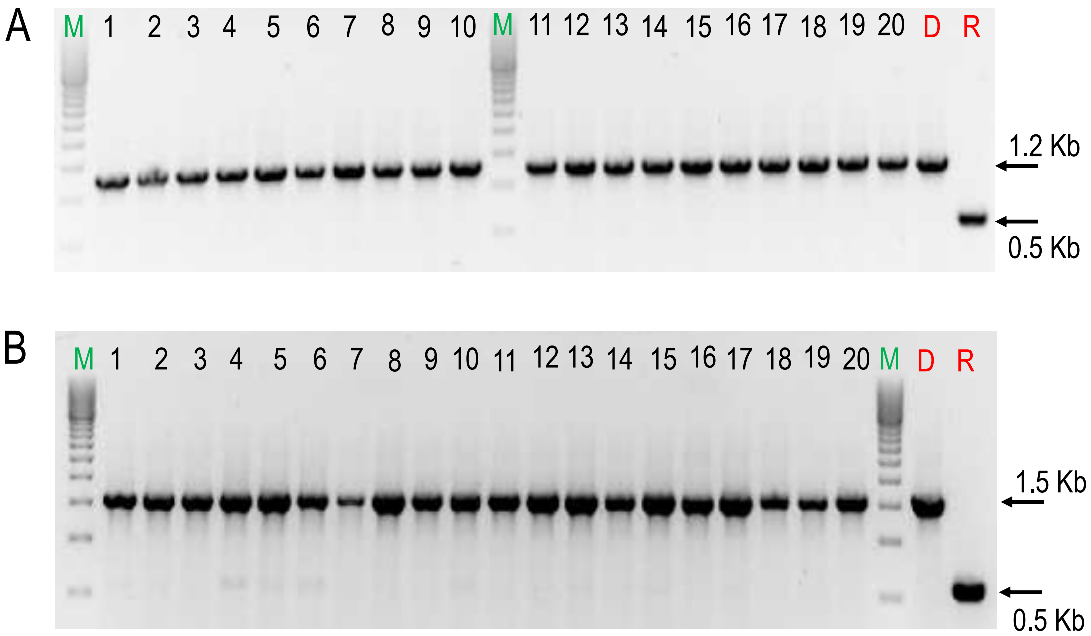

A. Twenty *P. putida* JS40 trans-conjugants (lanes 1 to 20) isolated in M9-Citrate-Gm_{50} media (*pyrF* transfer- selected across several experiments with donors *P. putida*-TRANS#9 and #18) were PCR checked with primers pyrF-F/pyrF-R. All PCRs showed the expected amplicon size for the intact *pyrF* gene (1.2 Kb), confirming the transfer of the marker gene. Control amplifications of #9 donor strain (D) and *P. putida* JS40 receptor strain (R) showed, respectively, the expected sizes of 1.2 Kb (wild-type gene) and 0.5 Kb (Δ*pyrF* deletion mutant). M stands for Bio-Rad EZ Load 500 bp Molecular Ruler. **B.** Twenty *P. putida* JS40 trans-conjugants (lanes 1 to 20) isolated in M9-Glucose-Ura-Gm_{50} media (*edd* transfer- selected across several experiments with donors *P. putida*-TRANS#9 and #18) were PCR checked with primers edd-check-F/edd-check-R. All PCRs showed the expected amplicon size for the intact *edd* gene (1.5 Kb), confirming the transfer of the marker gene. Control amplifications of #9 donor strain (D) and *P. putida* JS40 receptor strain (R) showed, respectively, the expected sizes of 1.5 Kb (wild-type gene) and 0.5 Kb (Δ*edd* deletion mutant). M stands for Bio-Rad EZ Load 500 bp Molecular Ruler.

SUPPLEMENTAL TABLES

Supplementary Table S1. Bacterial strains and plasmids used in this work.

Strain or plasmid	Relevant characteristics [a]	Reference or source
Escherichia coli		
CC118	Cloning host; Δ(*ara-leu*) *araD* Δ*lacX74 galE*, *galK phoA thiE1 rpsE*(Sp^{R}), *rpoB*(Rif^{R}), *argE*(Am), *recA1*	11
CC118λ*pir*	CC118, λ*pir* lysogen	12
DH5αλ*pir*	Cloning host for R6K-derived plasmids; *supE44*, Δ*lacU169* (φ*80 lacZ*ΔM15), *hsdR17* (*rk-mk*+), *recA1*, *endA1*, *thi1*, *gyrA*, *relA*, lysogenic λ*pir*	Lab collection
HB101	Helper strain used for conjugation; F^{-}, λ^{-}, *hsdS20* (rB^{-} mB^{-}), *rec*A13 *leuB6*(Am), *araC14* Δ(*gpt-pro*A)*62*, *lacY1 galK2*(Oc), *xyl-5*, *mtl-1*, *thiE1*, *rpsL20* (Sm^{R}), *glnX44* (AS)	13
TransforMax™ EC100D™ *pir* $^{+}$	F^{-} *mcr*A Δ(*mrr*-hsdRMS-*mcrBC*) (φ*80 lacZ*ΔM15) Δ*lac*X74 *rec*A1 *endA1 araD139* Δ(*ara*,*leu*)*7697 gal*U *galK* λ^{-} *rpsL nupG pir*+(DHFR).	Lucigen®
Pseudomonas putida		
EM42	KT2440 derivative; Δprophage1 Δprophage4 Δprophage3 Δprophage2 ΔTn*7* Δ*endA-1* Δ*endA-2* Δ*hsdRMS* Δflagellum ΔTn*4652*	4
TA280	EM42 derivative; *rpsL* (Sm^{R}), *rpoB* (Rif^{R}), gyrA (Nal^{R})	This work
TRANS#X	TA280 derivatives harboring mini-Tn5-TRANS insertions, Km^{R}, *rpsL* (Sm^{R}), *rpoB* (Rif^{R}), *gyrA* (Nal^{R})	This work
EM42 Δ*pyrF*Δ*edd*	EM42 derivative; Δ*pyrF*, Δ*edd*	6
JS40	EM42 derivative; Δ*pyr*F, Δ*edd,* mini-Tn*7* transposon harboring P$_{EM7}$-*gfp*, Gm^{R}	This work
Saccharomyces cerevisiae		
CRY1-2	MATα, *ura3*, Δ*cyh2R*	Lab collection

Plasmids		
pRK600	Helper plasmid used for conjugation; *oriV*(ColE1), RK2 (mob+ tra+); Cm^R	14
RP4	Wild type conjugative plasmid; Tra1-Tra2 Cores; *oriV*(RK2); Tc^R; Km^R; Ap^R	Lab Collection
pSEVA222S$_{\beta}$	Yeast shuttle vector; *oriV*(RK2); cargo [*lacZ*α-pUC19/I-SceI]; gadget β; Km^R	This work
pTRANS	Mini-Tn5-TRANS delivery plasmid; *oriV*(R6K); Ap^R; Km^R; CEN6-ARS209-URA3; TRANS module (Tra1-Tra2 Cores); *xylS*-P_m expression system	This work
pRS416	Yeast shuttle vector; *oriV*(ColE1); *lacZ*α; CEN6-ARS209; URA3; Ap^R	15
pSEVA221	Cloning vector; *oriV*(RK2); standard multiple cloning site; Km^R	10
pSEVA212S	Cloning vector; *oriV*(RK2); cargo [*lacZ*α-pUC19/I-SceI]; Km^R	10
pBAMD1-2	Mini-Tn5 delivery plasmid; *oriV*(R6K); Ap^R; Km^R	16
pSEVA238	Expression vector; *oriV*(pBBR1); cargo [*xylS*-P*m*]; standard multiple cloning site; Km^R	10
pTn7-M-PEM7-GFP	pTn7-M derivative with P_{EM7}-*gfp* in the mini-Tn*7* transposon; *oriV*(R6K); Km^R Gm^R	5
pTNS2	Helper plasmid for mini-Tn7 transposition; *oriV*(R6K), TnsABC+D specific transposition pathway; Ap^R	17

Supplementary Table S2. Primers, templates and PCR conditions of pSEVA222S$_\beta$ and pTRANS assemblies.

**pSEVA222S$_\beta$ Assembly**					
**PCR name**	**Primers**	**Template**	**Tm/Te**	**Size (bp)**	**Fragment amplified**
PCR-A	lacZa-AscI-F lacZa-AscI-R	pSEVA212S	66 ºC/ 30”	781	*lacZ*α-pUC19/I-SceI
PCR-B	CEN6-F/ CEN6-R	pRS416	57 ºC/ 30”	510	CEN6/ARS209
PCR-C	URA3-F/URA3-intR	pRS416	55 ºC/ 30”	219	URA3-partial
PCR-D	URA3-intF/ URA3-R	pRS416	55 ºC/ 30”	924	URA3-partial
PCR-E	URA3-F/ URA3-R	PCR-C+PCR-D	55 ºC/ 30”	1101	URA3 (cured of Pst-I restriction site)
PCR-F	RK2-Km-F/RK2-Km-R	pSEVA2214	60 ºC/ 2’	3440	*ori* RK2 and Km^R cassette
**pTRANS Assembly**					
**PCR name**	**Primers**	**Templates**	**Tm/Te**	**Size (bp)**	**Fragment amplified**
PCR-1	Tra1-F1 / Tra1-R1	RP4	60 ˚C / 2’	3995	Tra1-1
PCR-2	Tra1-F2 / Tra1-R2	RP4	60 ˚C / 2’	3852	Tra1-2
PCR-3	Tra2-F1 / Tra2-R1	RP4	60 ˚C / 2’	3766	Tra2-1
PCR-4	Tra2-F2 / Tra2-R2	RP4	60 ˚C / 2’	4106	Tra2-2
PCR-5	Tra2-F3 / Tra2-R3	RP4	61 ˚C / 2’	4064	Tra2-3
PCR-6	oriR6K-F/ oriR6K-R	pBAMD1-2	70 ˚C / 30”	586	*ori* R6K and ME-I
PCR7	pBAMD-F/ pBAMD-R	pBAMD1-2	60 ˚C / 2’	3799	TrpA, Km^R and Ap^R and ME-O
PCR-8	xylS-F / Pm-R	pSEVA258	60 ˚C / 1’	1987	*xylS*-P*m*
PCR-9	CEN6-F / URA3-R	pSEVA222S$_\beta$	50 ˚C / 1’	1611	CEN6/ARS209 and URA3
Hemi-Linker-1.1 (=PCR6)	oriR6K-F/ oriR6K-R	pBAMD1-2	70 ˚C / 30”	586	First half of Linker-1
Hemi-Linker-1.2	L1-F2 / L1-R2	RP4	60 ˚C / 30”	580	Second half of Linker-1
Hemi-Linker-2.1	L2-F1 / Tra1-R2	RP4	60 ˚C / 30”	511	First half of Linker-2
Hemi-Linker-2.2	L2-F2 / L2-R2	pSEVA258	60 ˚C / 30”	669	Second half of Linker2
Hemi-Linker-3.1	L3-F1 / Pm-R	pSEVA258	60 ˚C / 30”	581	First half of Linker-3
Hemi-Linker-3.2	L3-F2 / L3-R2	RP4	60 ˚C / 30”	546	Second half of Linker3
Hemi-Linker-4.1	L4-F1 / Tra2-R3	RP4	59 ˚C / 30”	631	First half of Linker-4
Hemi-Linker-4.2	L4-F2 / L4-R2	pBAMD1-2	60 ˚C / 30”	584	Second half of Linker-4
Hemi-Linker-5.1	L5-F1 / pBAMD-R	pBAMD1-2	60 ˚C / 30”	494	First half of Linker-5
Hemi-Linker-5.2	L5-F2 / L5-R2	pSEVA222S$_\beta$	60 ˚C / 30”	674	Second half of Linker-5
Linker-1	oriR6K-F / L1-R2	Linkers 1.1+1.2	60˚C / 45”	1169	Sewing PCR for Linker-1
Linker-2	L2-F1 / L2-R2	Linkers 2.1+2.2	60˚C / 45”	1160	Sewing PCR for Linker-2
Linker-3	L3-F1 / L3-R2	Linkers 3.1+3.2	60˚C / 45”	1079	Sewing PCR for Linker-3
Linker-4	L4-F1 / L4-R2	Linkers 4.1+4.2	60˚C / 45”	1192	Sewing PCR for Linker-4
Linker-5	L5-F1 / L5-R2	Linkers 5.1+5.2	60˚C / 45”	1147	Sewing PCR for Linker-5

Supplementary Table S3. Oligonucleotides used in this study.

Name	Sequence (5' → 3')[a]	Usage/Source[(b)]
lacZa-AscI-F	ggatctcaagaagatcctttgatcttttctacGGCGCGCCCAGCTGTCTAG	To amplify the *lacZ*α-pUC19/I-SceI cargo from pSEVA212S; homology tail with RK2-Km-R
lacZa-AscI-R	attttaaattataattatttttatagcacgtgatgaaaagGGGACCCCTGGATTCTCACC	To amplify the *lacZ*α-pUC19/I-SceI cargo from pSEVA212S; homology tail with CEN6-F
CEN6-F	cttttcatcacgtgctataaaaataattataatttaaaatTTTTAATATAAATATATAAATTAAAAATAGAAAG	To amplify the CEN6/ARS209 cassette from pRS416, removing a Swa-I site; homology tail with lacZa-AscI-R
CEN6-R	GATCGCTTGCCTGTAAC	To amplify the CEN6/ARS209 cassette from pRS416
URA3-F	taaaagatacgaggcgcgtgtaagttacaggcaagcgatcCCTTTTCAATTCATCATTTTTTTTTTATTC	To amplify the URA3 cassette from pRS416; homology tail with CEN6-R
URA3-intR	GATTTATCTTCGTTTCCTGCAATTTTTGTTCTGTGCAGTTGG	To amplify the URA3 cassette from pRS416 removing a Pst-I restriction site
URA3-intF	CCAACTGCACAGAACAAAAATTGCAGGAAACGAAGATAAATC	To amplify the URA3 cassette from pRS416, removing a Pst-I restriction site
URA3-R	tgcaatgtaacatcagagattttgagacacaaatttaaatGGGTAATAACTGATATAATTAAATTGAAG	To amplify the URA3 cassette from pRS416; homology tail with RK2-Km-F
RK2-Km-F	ATTTAAATTTGTGTCTCAAAATCTCTGATG	To amplify the RK2 origin of replication and Km^R cassette from pSEVA2214
RK2-Km-R	GGCGCGCCGTAGAAAAGATCAAAGGATCTTC	To amplify the RK2 origin of replication and Km^R cassette from pSEVA2214
Tra1-F1	ATATCCCCCTACCCTCACC	To amplify the Tra1-1 region from RP4
Tra1-R1	GAAACGGCTTCATCTTCGAG	To amplify the Tra1-1 region from RP4
Tra1-F2	GATCCGCTCCTTGAACTCTG	To amplify the Tra1-2 region from RP4
Tra1-R2	TCAAACAAGCCCAGCTAACG	To amplify the Tra1-2 region from RP4 and Linker-2
Tra2-F1	TTGCCTGTGCGATAATGTTGC	To amplify the Tra2-1 region from RP4
Tra2-R1	CCGAGGTTCATCAGCTCTTC	To amplify the Tra2-1 region from RP4
Tra2-F2	TCGGCCACACCTTTATGTTC	To amplify the Tra2-2 region from RP4
Tra2-R2	GAGTTGATGCCCGAGATAAGC	To amplify the Tra2-2 region from RP4

Tra2-F3	CATGGGTTCGGAAGGAGTAAG	To amplify the Tra2-3 region from RP4
Tra2-R3	CGCTTTTTTCAGTTGCATGGTC	To amplify the Tra2-3 region from RP4 and Linker-4
oriR6K-F	ttatagcacgtgatgaaaagGGCCGGCCGATC TGAAGATCAGCAGTTCAAC	To amplify the *ori* R6K and ME-I region from pBAMD1-2, homology tail with CEN6-F. Also for Linker-3
oriR6K-R	GAATTCGCGCGGCCGCGG	To amplify the *ori* R6K and ME-I region from pBAMD1-2. Also for Linker-3
pBAMD-F	CTGCAGGCATGCAAGCTTG	To amplify *trpA* gene, Km^R and Ap^R cassettes and ME-O region from pBAMD1-2
pBAMD-R	GAAAAGGACAAACGCGTGTCC	To amplify *trpA* gene, Km^R and Ap^R cassettes and ME-O region from pBAMD1-2. Also for Linker-5
xylS-F	ACGTTCGTAATCAAGCCACTTC	To amplify the *xylS*-P_m cassette from pSEVA258
Pm-R	CATGGTCATGACTCCATTATTATTGTT TC	To amplify the *xylS*-P_m cassette from pSEVA258 and Linker-3
L1-F2	ccgcggccgcgcgaattcATATCCCCCTACC CTCACC	To amplify the Linker-1 to connect the *ori* R6K region with Tra1-1 region, homology tail with oriRK6-R
L1-R2	GAGGGGATCACGATATGAGC	To amplify the Linker-1 to connect the *ori* R6K region with Tra1-1 region
L2-F1	TGCTATGAGCGACCAGATTG	To amplify the Linker-2 to connect Tra1-2 region with the *xylS*-P_m cassette
L2-F2	cgttagctgggcttgtttgaACGTTCGTAATCAA GCCACTTC	To amplify the Linker-2 to connect Tra1-2 region with the *xylS*-P_m cassette, homology tail with Tra1-R2
L2-R2	CAAGCCGACCTGACCTATTC	To amplify the Linker-2 to connect Tra1-2 region with the *xylS*-P_m cassette
L3-F1	TCCATGAGCAAACTGAAACG	To amplify the Linker-3 to connect the *xylS*-P_m cassette with Tra2-1
L3-F2	gaaacaataataatggagtcatgaccatgTTGCCT GTGCGATAATGTTGC	To amplify the Linker-3 to connect the *xylS*-P_m cassette with Tra2-1, homology tail with Pm-R
L3-R2	GCCAATGACGAGGATGTTTC	To amplify the Linker-3 to connect the *xylS*-P_m cassette with Tra2-1
L4-F1	AGGCCAGCGATAACGTCTC	To amplify the Linker-4 to connect Tra2-3 with the TrpA, Km^R and Ap^R region of pBAMD1-2

L4-F2	gaccatgcaactgaaaaaagcgCTGCAGGCA TGCAAGCTTG	To amplify the Linker-4 to connect Tra2-3 with the TrpA, Km^R and Ap^R region of pBAMD1-2, homology tail with Tra2-R3
L4-R2	TATGCAGACGACGCAGAAAC	To amplify the Linker-4 to connect Tra2-3 with the TrpA, Km^R and Ap^R region of pBAMD1-2
L5-F1	GAAAGAACTGACCGCGTTTC	To amplify the Linker-5 to connect TrpA, Km^R and Ap^R region of pBAMD1-2 with the CEN6/ARS209 cassette
L5-F2	ggacacgcgtttgtccttttcGGGTAATAACTGA TATAATTAAATTGAAGCTC	To amplify the Linker-5 to connect TrpA, Km^R and Ap^R region of pBAMD1-2 with the CEN6/ARS209 cassette, homology tail with pBAMD-R
L5-R2	TAAAGGCATTATCCGCCAAG	To amplify the Linker-5 to connect TrpA, Km^R and Ap^R region of pBAMD1-2 with the CEN6/ARS209 cassette
MEO-Km-extF	CGTCTGTTTCAGAAATATGGCAT	Used in arbitrary PCR round 1 for Tn-5 localization [2]
ARB6	GGCACGCGTCGACTAGTACNNNNNNN NNNNACGCC	Used in arbitrary PCR round 1 for Tn-5 localization [2]
MEO-Km-intF	ATCTGATGCTGGATGAATTTTTC	Used in arbitrary PCR round 2 for Tn-5 localization [2]
ARB2	GGCACGCGTCGACTAGTAC	Used in arbitrary PCR round 2 for Tn-5 localization [2]
SR	G*T*C*A*GACGCACACGGCATACTTTA CGCAGTGCCGAGTTAGGTTTT**G**TCGG CGTGGTGGTGTACACACGGGTGCAC ACGCCACGACGCTGC	Recombineering oligo for *rpsL* gene: AAA (K43) changed to ACA (T43), mismatch A:G, confers Sm resistance [18]
rpsL-Fw	GACATGAAATGTTGCCGATG	To amplify and sequence part of *rpsL* gene of *P. putida* [19]
rpsL-Rv	CTGTTCTTGCGTGCTTTGAC	To amplify and sequence part of *rpsL* gene of *P. putida* [19]
RR	TCCGAGAGAGGGTTGTTCTGGTCCAT GAAC**A**GGGACAGCTGGCTGGAACCG AAGAACTCT	Recombineering oligo for *rpoB* gene: CAG (Q518) changed to CTG (L518), mismatch A:A, confers Rif resistance [5]
rpoB-F	CCTGGGTAACCGTCGCGTACGGTG	To amplify and sequence part of *rpoB* gene of *P. putida* [5]
rpoB-R	CGCCTTCCTTCACCACGCGGTACG	To amplify and sequence part of *rpoB* gene of *P. putida* [5]
NR	AACGAGAACGGCTGGGCCATACGCA CGATGGT**A**T**T**GTAGACCGCAGTGTCG CCGTGCGGGTGGTA	Recombineering oligo for *gyrA* gene: GAC (D87) changed to AAT (N87), mismatches G:T

		and C:A, confers Nal resistance [18]
gyrA-F	GGCCAAAGAAATCCTCCCGGTCAA	To amplify and sequence part of *gyrA* gene of *P. putida* [18]
gyrA-R	AGCAGGTTGGGAATACGCGTCG	To amplify and sequence part of *gyrA* gene of *P. putida* [18]
pyrF-F	CGAGGGCTATGATGAGTATC	To amplify and sequence the *pyrF* gene (PP_1815) of *P. putida* [20]
pyrF-R	GTCAGGTGAAGAGCAAAGAG	To amplify and sequence the *pyrF* gene (PP_1815) of *P. putida* [20]
edd-check-F	TAAACCGCCCTTACAATTAG	To amplify and sequence the *edd* gene (PP_1010) of *P. putida* EM42 [6]
edd-check-R	ACCAACGCAACCTTGTAG	To amplify and sequence the *edd* gene (PP_1010) of *P. putida* EM42 [6]

(a) Lower case denotes homology tails with other oligos. Ns in primer ARB6 are degenerated positions (N=A,G,CT). Mutated Pst-I site (CTGCAA/TTGCAG) appears in red in primers URA3-intR/ URA3-intF and the mutated position is underlined. In CEN6-F, mutated Swa-I site (atttaaaa) appears in blue and the mutated position is underlined. Asterisks denote phosphorothioate bonds. Single changes introduced by recombineering oligonucleotides SR, NR and RR are highlighted in bold

(b) A reference source only appears for primers designed in other works.

SUPPLEMENTAL REFERENCES

(1) Sambrook, J., Fritsch, E. F., and Maniatis, T., (1989) Molecular cloning: a laboratory manual. Cold Spring Harbor Laboratory Press: Cold Spring Harbor, NY

(2) Martinez-Garcia, E., Aparicio, T., de Lorenzo, V., and Nikel, P. I. (2017) Engineering Gram-Negative Microbial Cell Factories Using Transposon Vectors. *Methods Mol Biol 1498*, 273-293.

(3) Martinez-Garcia, E., and de Lorenzo, V. (2012) Transposon-based and plasmid-based genetic tools for editing genomes of gram-negative bacteria. *Methods Mol Biol 813*, 267-283.

(4) Martinez-Garcia, E., Nikel, P. I., Aparicio, T., and de Lorenzo, V. (2014) *Pseudomonas 2.0*: genetic upgrading of *P. putida* KT2440 as an enhanced host for heterologous gene expression. *Microb Cell Fact 13*, 159.

(5) Aparicio, T., Nyerges, A., Martinez-Garcia, E., and de Lorenzo, V. (2020) High-Efficiency Multi-site Genomic Editing of *Pseudomonas putida* through Thermoinducible ssDNA Recombineering. *iScience 23*, 100946.

(6) Aparicio, T., de Lorenzo, V., and Martinez-Garcia, E. (2019) CRISPR/Cas9-enhanced ssDNA recombineering for *Pseudomonas putida*. *Microb Biotechnol 12*, 1076-1089.

(7) Lambertsen, L., Sternberg, C., and Molin, S. (2004) Mini-Tn7 transposons for site-specific tagging of bacteria with fluorescent proteins. *Environmental Microbiology 6*, 726-732.

(8) Muller, H., Annaluru, N., Schwerzmann, J. W., Richardson, S. M., Dymond, J. S., Cooper, E. M., Bader, J. S., Boeke, J. D., and Chandrasegaran, S. (2012) Assembling large DNA segments in yeast. *Methods Mol Biol 852*, 133-150.

(9) Ouwerkerk, P. B. F., and Meijer, A. H. (2001) Yeast One-Hybrid Screening for DNA-Protein Interactions. *Current Protocols in Molecular Biology 55*, 12.12.11-12.12.12.

(10) Silva-Rocha, R., Martinez-Garcia, E., Calles, B., Chavarria, M., Arce-Rodriguez, A., de Las Heras, A., Paez-Espino, A. D., Durante-Rodriguez, G., Kim, J., Nikel, P. I., Platero, R., and de Lorenzo, V. (2013) The Standard European Vector Architecture (SEVA): a coherent platform for the analysis and deployment of complex prokaryotic phenotypes. *Nucleic Acids Res 41*, D666-675.

(11) Grant, S. G., Jessee, J., Bloom, F. R., and Hanahan, D. (1990) Differential plasmid rescue from transgenic mouse DNAs into *Escherichia coli* methylation-restriction mutants. *Proc Natl Acad Sci USA 87*, 4645-4649.

(12) Herrero, M., de Lorenzo, V., and Timmis, K. N. (1990) Transposon vectors containing non-antibiotic resistance selection markers for cloning and stable chromosomal insertion of foreign genes in gram-negative bacteria. *J Bacteriol 172*, 6557-6567.

(13) Boyer, H. W., and Roulland-Dussoix, D. (1969) A complementation analysis of the restriction and modification of DNA in *Escherichia coli*. *J Mol Biol 41*, 459-472.

(14) Kessler, B., de Lorenzo, V., and Timmis, K. N. (1992) A general system to integrate lacZ fusions into the chromosomes of gram-negative eubacteria: regulation of the Pm promoter of the TOL plasmid studied with all controlling elements in monocopy. *Mol Gen Genet 233*, 293-301.

(15) Sikorski, R. S., and Hieter, P. (1989) A system of shuttle vectors and yeast host strains designed for efficient manipulation of DNA in *Saccharomyces cerevisiae*. *Genetics 122*, 19-27.

(16) Martinez-Garcia, E., Aparicio, T., de Lorenzo, V., and Nikel, P. I. (2014) New transposon tools tailored for metabolic engineering of gram-negative microbial cell factories. *Front Bioeng Biotechnol 2*, 46.

(17) Choi, K. H., Gaynor, J. B., White, K. G., Lopez, C., Bosio, C. M., Karkhoff-Schweizer, R. R., and Schweizer, H. P. (2005) A Tn7-based broad-range bacterial cloning and expression system. *Nat Methods 2*, 443-448.

(18) Aparicio, T., Nyerges, A., Nagy, I., Pal, C., Martinez-Garcia, E., and de Lorenzo, V. (2020) Mismatch repair hierarchy of *Pseudomonas putida* revealed by mutagenic ssDNA recombineering of the *pyrF* gene. *Environ Microbiol 22*, 45-58

(19) Ricaurte, D. E., Martinez-Garcia, E., Nyerges, A., Pal, C., de Lorenzo, V., and Aparicio, T. (2018) A standardized workflow for surveying recombinases expands bacterial genome-editing capabilities. *Microb Biotechnol 11*, 176-188.

(20) Aparicio, T., Jensen, S. I., Nielsen, A. T., de Lorenzo, V., and Martinez-Garcia, E. (2016) The Ssr protein (T1E_1405) from *Pseudomonas putida* DOT-T1E enables oligonucleotide-based recombineering in platform strain *P. putida* EM42. *Biotechnol J 11*, 1309-1319.
